# Knowledge of HIV-related disabilities and challenges in accessing care: Qualitative research from Zimbabwe

**DOI:** 10.1371/journal.pone.0181144

**Published:** 2017-08-09

**Authors:** Lena Morgon Banks, Maria Zuurmond, Rashida Ferrand, Hannah Kuper

**Affiliations:** 1 International Centre for Evidence in Disability, London School of Hygiene & Tropical Medicine, London, United Kingdom; 2 Clinical Research Department, London School of Hygiene & Tropical Medicine, London, United Kingdom; 3 Biomedical Research and Training Institute, Harare, Zimbabwe; SOUTH AFRICA

## Abstract

**Introduction:**

While the rapid expansion in antiretroviral therapy access in low and middle income countries has resulted in dramatic declines in mortality rates, many people living with HIV face new or worsening experiences of disability. As nearly 1 in 20 adults are living with HIV in sub-Saharan Africa–many of whom are likely to develop disabling sequelae from long-term infection, co-morbidities and side effects of their treatment–understanding the availability and accessibility of services to address HIV-related disabilities is of vital importance. The aim of this study thus is to explore knowledge of HIV-related disabilities amongst stakeholders working in the fields of HIV and disability and factors impacting uptake and provision of interventions for preventing, treating or managing HIV-related disabilities.

**Methods:**

In-depth, semi-structured interviews were conducted with ten stakeholders based in Harare, Zimbabwe, who were working in the fields of either disability or HIV. Stakeholders were identified through *a priori* stakeholder analysis. Thematic Analysis, complemented by constant comparison as described in Grounded Theory, was used to analyse findings.

**Results:**

All key informants reported some level of knowledge of HIV-related disability, mostly from observations made in their line of work. However, they reported no interventions or policies were in place specifically to address HIV-related disability. While referrals between HIV and rehabilitation providers were not uncommon, no formal mechanisms had been established for collaborating on prevention, identification and management. Additional barriers to accessing and providing services to address HIV-related disabilities included: the availability of resources, including trained professionals, supplies and equipment in both the HIV and rehabilitation sectors; lack of disability-inclusive adaptations, particularly in HIV services; heavy centralization of available services in urban areas, without accessible, affordable transportation links; and attitudes and understanding among service providers and people living with HIV-related disabilities.

**Conclusions:**

As people living with HIV are surviving longer, HIV-related disabilities will become a major source of disability globally, particularly in sub-Saharan Africa where infection is endemic. Preventing, treating and managing HIV-related disabilities must become a key component of both HIV response efforts and rehabilitation strategies.

## Introduction

The advent and roll-out of antiretroviral therapy (ART) has changed the prognosis of HIV from one of inescapable death to a chronic, yet manageable, condition [1]. With coverage of ART and other treatment supports increasing even in low and middle income countries (LMICs)[2], many of the estimated 35 million people living with HIV globally [3] can expect to live significantly longer, even to a near-normal life expectancy[4, 5]. While ART decreases the risk of death and development of opportunistic infections, many people living with HIV face new or worsening experiences of disability [6, 7].

People with disabilities include those who have long-term physical, mental and intellectual or sensory impairments which in interaction with various barriers may hinder their full and effective participation in society on an equal basis with others [8]. HIV infection, its co-morbidities and side effects of their treatment may lead to the development of a range of impairments (e.g. musculoskeletal impairment) [6, 9]. These impairments in turn may limit the performance of activities (e.g. self-care, mobility) and consequently restrict participation in society (e.g. employment, education). The conceptualisation of disability therefore incorporates the presence of impairments, activity limitations and participation restriction, as well as contextual factors that may mitigate or exacerbate disability (e.g. access to healthcare). Disability is an important issue—the World Report on Disability estimated that the 1 billion people living with disabilities globally are among the poorest and most marginalised groups [10]. Services needed to alleviate disability range from prevention or treatment of impairment to providing rehabilitation or inclusive services, but are usually inadequate, particularly in low and middle income countries [10].

HIV-related disabilities are increasingly being recognised as a pressing, but underexplored public health and development concern [1, 6, 9]. The implications of HIV-related disabilities has particular importance for sub-Saharan Africa, which accounts for 70% of the global burden of HIV and where nearly 1 in 20 adults are living with HIV [3]. In a systematic review of HIV-related disabilities in this region, prevalence of disabilities amongst people living with HIV was high across a range of domains–including sensory, cognitive and physical impairment as well as general functioning [9]. Prevalence varied based on impairment type, from a median of 11% for visual impairment to 40% for cognitive impairment in adults. Developmental delay in particular was highly prevalent–affecting over two-thirds of infants living with HIV—indicating significant detrimental effects of HIV on neurodevelopment, which may have long-term consequences across these children’s life-courses [9]. Across studies, risk of disability was significantly higher when compared to an HIV-uninfected comparator.

Evidence on HIV-related disabilities is increasingly being translated into advocacy and programmatic actions by international actors, with engagement from some regional partners in sub-Saharan Africa [11–13]. This includes the creation of e-modules for rehabilitation providers on the role of rehabilitation for people living with HIV [14], as well as increasing engagement of Disabled Peoples’ Organisations (DPOs) in HIV responses [15, 16]. However, less is known about how widely this information has been filtered down across the range of practitioners working in the fields of HIV and disability at a national or local level–from care providers to policymakers–and how this knowledge is operationalised into local policy and practice so that people living with HIV are able to access the needed care to prevent, treat and manage HIV-related disabilities.

We therefore conducted a qualitative study in Harare, Zimbabwe amongst stakeholders working in the fields of either HIV or disability to explore their knowledge of HIV-related disabilities and on the availability and accessibility of interventions for preventing, treating or managing HIV-related disabilities.

### HIV & disability in Zimbabwe: Background on the study setting

This research took place in Harare, the capital of Zimbabwe. At almost 15%, Zimbabwe has the fifth highest prevalence of HIV in sub-Saharan Africa [17, 18]. Incidence of new infections, as well as progression to AIDS and AIDS-related illnesses has been steadily declining due to the successful implementation of a range of HIV prevention and treatment programmes, including rigorous enforcement of prevention of mother to child transmission (PMTCT), behaviour change activities, and free provision of ART [17, 19]. National all-age coverage of ART is 51% as of 2014, which is similar to other Southern African countries [20].

Concerning rehabilitation and disability supports, Zimbabwe was a leader in advancing the disability movement in southern Africa the 1970s and 80s, including in the provision of rehabilitation and other social services for people with disabilities [21, 22]. However, economic decline and political unrest in recent decades have eroded many of these gains [21]. Still, the public rehabilitation system, which was one of the first national systems in sub-Saharan Africa, is relatively well organised compared to neighbouring countries [22]. The entry point of the rehabilitation system are the district-level central rehabilitation units (CRU), which provide assessments of disability. If deemed to have a disability by the CRU, individuals are entitled to receive basic services and some medications free of charge; however, assistive devices and more complex care must typically be paid for out-of-pocket. After receiving a diagnosis at the CRU, individuals may then be referred to local centres for ongoing care, or for complex cases, continue receiving care at the CRU. Most individuals come into contact with the CRU through referrals from other health providers, which are typically performed on an ad hoc basis; however standardised protocols for primary care providers to detect developmental delay in young children were being introduced at the time of the study.

There has been increasing attention to the link between HIV and disability in Zimbabwe, albeit mostly in that people with disabilities are at increased risk for HIV infection rather than of HIV leading to disability. Notably, the 2015–2018 National HIV/AIDS Strategic Plan, which was being drafted at the time of the study, acknowledges a link between disability and HIV, which was not included in the previous 2011–2015 Plan [23, 24] Specifically, the 2015–2018 Plan notes that people with disabilities are at increased risk of HIV infection and thus seeks greater disability-inclusion in prevention and treatment programmes. Further, the Plan seeks to take a rights-based approach to promote the dignity and non-discrimination of people living with HIV, with special emphasis for “people with disabilities…and others who are socially excluded” [24]. Civil society has also played a role in promoting greater awareness of links between HIV and disability. For example, the Disability, HIV & AIDS Trust (DHAT) is a regional non-profit organisation operational in Southern Africa with headquarters in Harare; it is focused primarily on advocacy and awareness raising among policymakers, HIV and AIDS organisations and people with disabilities themselves about the need to greater disability-inclusion within HIV and AIDS responses [15].

Within this context, there is a growing interaction between the HIV and disability communities. While there is a greater focus in policy and advocacy on people with disabilities being at heightened vulnerability for both HIV infection and exclusion from HIV care and treatment, it is less apparent whether awareness of HIV as a cause or contributors to disability is as widespread. Given the emergence of linkages between disability and HIV, Zimbabwe–particularly Harare where most national decision-making occurs–presents a novel setting to better understand awareness of and interventions for HIV-related disability among key stakeholders.

## Methods

### Study sample

The target population of this research was stakeholders working at the national or local level in Zimbabwe in the fields of HIV and AIDS and/or disability. Participants included representatives from government, the health sector as well as national and international non-governmental organisations and Disabled Peoples’ Organisations, who were engaged in a range of programmatic, advocacy or policy activities.

Purposive of sampling was employed to recruit participants. Participants were identified through *a priori* stakeholder analysis conducted by researchers at the London School of Hygiene & Tropical Medicine (MZ and HK) and Africaid Zvandiri (unpublished), with snowball sampling employed to identify additional individuals.

A total of ten participants were included in the final sample. Four participants specialized primarily in HIV and AIDS and four in disability. An additional two had experience in both areas, namely in promoting greater inclusion of people with disabilities in HIV prevention and treatment activities. Individuals represented organizations involved in the following domains: rehabilitation provision (n = 3), HIV service provision (n = 2), HIV advocacy (n = 3), disability advocacy (n = 3) and programme funding/technical assistance (n = 3). All participants were based in Harare, Zimbabwe although the majority were working on programmes operating outside of the capital as well. Most participants were Zimbabwean, while three were expatriates from neighbouring countries (n = 2) or outside Africa (n = 1), though all had worked in Zimbabwe for several years. Most of the sample (n = 6) was female. All participants were proficient in English, which is not atypical for professionals working in Harare, where English proficiency is high and required for most jobs,

### Data collection

Data collection was conducted in August-September 2013 using in-depth semi-structured interviews. Topic guides were developed with input from all authors. Key topics covered in interviews included knowledge of links between HIV and disability, how this knowledge was operationalized in relevant programmes and policies in Zimbabwe and barriers to the provision and uptake of services for preventing, treating or managing HIV-related disabilities.

All interviews were conducted in English and led by the lead researcher (LMB). Detailed notes were taken during interviews, which were supplemented through transcriptions of interview recordings.

### Ethical considerations

Verbal consent was obtained from all participants at the start of each interview. As part of the informed consent process, all participants were given a written information sheet detailing the study objectives and their rights as participants. These details were also summarised orally by the lead researcher and participants were given time to ask questions. Verbal consent rather than written consent was specified in the application for ethical approval as it was anticipated that some of the interviews would be phone-based, though as all of participants were available for in-person meetings, all were conducted in person.

Ethical approval was obtained from the London School of Hygiene & Tropical Medicine in July, 2013. Ethics approval in Zimbabwe was not required at the time of the study, as the interviews focused on professional opinions/expertise rather than the collection of personal data.

### Data analysis

Thematic Analysis, complemented by constant comparison as described in Grounded Theory, was used to analyse findings through the following steps [25, 26]. First, to become familiar with the data, the lead researcher (LMB) listened to interview recordings and re-read detailed interview notes and transcripts, both during and at the end of data collection. Inductive, open coding of notes/transcripts then was used to identify important features in the data. [26]. Codes were then collated into themes and sub-themes, which were reviewed by an additional researcher (MZ), who has expertise in qualitative health research.

To explore these perceptions on challenges in the provision, delivery and uptake of services to address HIV-related disability, codes and themes from the data on this topic were then interpreted in the context of Peters et al’s framework on determinants of access to healthcare in developing countries [27]. Through this process, the study’s findings on this topic could be contextualised in light of existing research in barriers to health service access.

### Positionality

All interviews were conducted by the lead researcher (LMB), who is a female of Canadian background. At the start of the interviews, the researcher introduced herself as a public health Masters student at the London School of Hygiene & Tropical Medicine who was conducting this research as part of her dissertation. Prior to commencing the study, the researcher had had experiencing working in the fields of both HIV and AIDS and disability separately and had recently begun to explore their interrelationship through a systematic review on HIV-related disability [9].

The background of the lead researcher could have implications on the dynamics of the interviews, as well as in the interpretation of data. For example, since the researcher was an outsider in Zimbabwe with no affiliation to any local groups, organisations or political parties, respondents may have viewed her as unbiased and were thus more open in their responses. However, as someone new to working in Zimbabwe, certain contextual details may have been overlooked in the analysis of the data. As a check against this potential bias, the results were reviewed by a researcher (RF) who has lived and worked in Zimbabwe for 15 years and has extensive experience in HIV research, including in disability and other chronic complications from HIV.

## Results

All key informants reported some level of knowledge of HIV-related disabilities, mostly from observations made in their line of work rather than through access to formal training or access to educational materials on the topic. Although the majority indicated that more research was needed in this field and that it was difficult to disentangle whether the links they had observed between HIV and certain impairments were causal, all suspected that HIV could at least contribute to the development or exacerbation of certain impairments. This opinion was rationalized through observations such as: certain impairments disproportionately affecting people living with HIV; impairments developing following an opportunistic infection targeted to the affected body structure; delays in initiating ART, lags in adherence and disease progression increasing likelihood of developing or worsening an impairment; and ART initiation and continuous adherence leading to improvements in functioning.

However, participants noted that awareness about the links between HIV and disability was limited amongst other individuals and programmes working in these fields in Zimbabwe. Furthermore, to their knowledge, no interventions or policies were in place specifically to address HIV-related disability. Some stakeholders noted anecdotally of referrals between HIV and rehabilitation providers, but these were done on an ad hoc basis and mostly for more severe forms of impairment. No participant noted any formal mechanisms for collaboration on prevention, identification and management of HIV-related disabilities between HIV and rehabilitation sectors. Given the lack of specialised services or coordinated action for HIV-related disability, most people living with HIV-related disability needed to navigate HIV, rehabilitation and other health services separately in an attempt to receive needed care.

Challenges in the provision, delivery and uptake of services to address HIV-related disability are grouped according to the determinants of access outlined in Peters et al’s framework of (1) availability, (2) geographic accessibility, (3) financial accessibility, and (3) acceptability.

### Availability of services

Lack of adequate resources was cited universally as a primary barrier to the provision of services. Both HIV and rehabilitation services–particularly the latter–face shortfalls in availability of key inputs such as skilled professionals, supplies and equipment. Furthermore, increasing coordination between HIV and disability services, which although deemed essential for addressing HIV-related disabilities, would lead to additional demands on an already overburdened health system, at least in the early stages of programme development and initiation. This challenge was voiced particularly strongly by individuals working primarily on the HIV side. Although recognizing the importance of mainstreaming disability into their programmes, staff feared that they did not have the time and resources to make the necessary adaptations:

“Health workers are already overburdened in the things they’ve already been asked to do lately—constantly tasked with another thing to look for, to do—first it’s testing, it’s male circumcision, PMTCT, then its adherence support, now it’s sexual and reproductive health now we’re talking about looking for disability as well…but it’s a very resource constrained setting.” [HIV service provider]

Due to this lack of specialised or coordinated care for HIV-related disability, people with HIV-related disabilities are left to independently navigate the separate HIV and rehabilitation systems to receive needed care. Within these separate systems, challenges to access persist. Notably, the inaccessibility of healthcare facilities and the lack of accommodations was highlighted as a barrier to access–namely to HIV services–for people with certain impairments. For example, the lack of alternative forms of communication (e.g. Braille, sign language, pictorial forms) was listed as a major challenge to the delivery of important information and the participation of individuals with sensory or intellectual impairments in their own care–important given the strong link between HIV and cognitive impairment [9]. These barriers to accessing timely, quality HIV services may cause irregular access to ART or inadequate treatment for opportunistic infections, all of which can lead to or exacerbate disability.

### Geographic accessibility

Compounded with the lack of coordinated or specialised care for HIV-related disabilities, when seeking services through the siloed HIV or rehabilitation sectors, what limited resources are available for rehabilitation and HIV care are heavily centralized. As previously mentioned, the CRU is the collection site where all individuals are referred for assessment and diagnosis. Once a diagnosis is made, most people are referred to local centres for ongoing support. However, availability of local centres for ongoing case management is limited: while Harare has fourteen local centres, most rural districts only have the one CRU. Additionally, rehabilitative services and resources available in rural district CRUs tend to be much more limited than those offered by more central CRUs. Many key informants noted that the centralization of rehabilitation services leads to barriers in accessing care, particularly for individuals living in rural areas:

“There’s rehab units in every district but some communities, for them to get to the district [centre] it can be something like 200 km–you can’t go and come back the same day, you have to stay overnight. In rural areas, [there’s] still a lot of challenges in terms of personnel, transport, equipment. That’s one of the biggest challenges which I think the nation, government is still facing to support outreach activities by rehab staff so that they can go out to the communities.” [Disability specialist and rehabilitation provider]

For HIV services, although more widely available for adults even in rural areas, paediatric services are lagging. At the time of data collection, there was only one lab in the country that could test infants (<18 months), leading to long delays in receiving a diagnosis and subsequently accessing services–a known risk factor for HIV-related developmental delay. Furthermore, ART coverage for children remains low, due to challenges such as a limited number of health facilities equipped to deliver paediatric ART.

Compounded with the centralization of services, nearly all respondents indicated that individuals living with HIV and disability faced additional challenges that complicated physical access to services. Mobility limitations combined with inaccessible transportation or the need for assistance were commonly cited barriers. Further, people living with both disability and HIV, many noted, may be more likely to be geographically isolated from needed services as a consequence of the marginalization arising from stigma from HIV and/or disability:

“People are unwilling to rent rooms if family has child with disability and/or HIV…so often they send a child [with HIV and/or disability] to rural homes to live with family so that parents can work. Seeking care [there] can be difficult, delayed because you don’t have your mother, your father; you’re staying with your grandmother and then it’s such a hassle to get you to seek care unless you’re really, really sick.” [iNGO, specialist in child protection]

### Financial accessibility

Once diagnosed with either HIV or a disability, an individual is entitled to free basic rehabilitation/HIV services and certain drugs (including ART) through the public system. However, other expenses, such as for additional services and supports (including assistive devices), transportation to and from appointments, and opportunity costs of time forgone with appointments or caring for an individual with HIV and disability, are out of pocket. The magnitude of these costs can be substantial, given the extent of care required for management of both HIV and disability and considering that many individuals and families have a low capacity to pay:

“The mother is often dealing with HIV [herself], struggling financially…mostly have single mothers, since fathers often divorce if the child has a disability or HIV…and then there’s a child with a need for both disability and HIV services.” [iNGO, specializing in HIV advocacy]

Similarly, cost–or expectations of cost–was cited by most respondents as the main reason why specific services or programmes for addressing HIV-related disabilities–outside of the standard package of services in general rehabilitation and HIV sectors–were not being offered.

“The other concern we have is—and this is a big area of concern—once we diagnose all these children [with HIV] with a disability and my feeling is that there could be quite a large number, we then ethically have to be able to respond… But then they need all manner of different devices which the cost is quite high. The range of interventions could be very broad.” [NGO, advocacy and support for children and adolescents with HIV]

### Acceptability

Acceptability can be defined as “the relationship of clients’ attitudes about personal and practice characteristics of providers to the actual characteristics of existing providers, as well as to provider attitudes about acceptable personal characteristics of clients”[28].

A key challenge, noted particularly by key informants working in rehabilitation, surrounded the acceptability of available services to potential users. When individuals lacked a holistic understanding of both the nature and causes of their condition, seeking needed care may be delayed due to misattribution of symptoms of HIV and disability to other causes. For example, disability and HIV is often believed to be caused by witchcraft, leading families to first seek traditional rather than biomedical care; similarly, if a person is first identified as disabled, symptoms of HIV may be viewed as part of their disability.

Furthermore, even if care is sought, the mismatch between high expectations of the benefits from treatment/rehabilitation and reality of the long-term, complex care often needed could lead to discouragement:

“People have expectations that rehab will cure disability… When parents first come with their child they are highly motivated, they are very optimistic. [But] therapy [for a child with HIV-related disability] can take longer, the child can get an OI [opportunistic infection] and regress…and then they start from square one or further back. [This] can create frustration and depression especially on the part of the parent/caregiver because they see their child making these gains and then they regress.” [Rehabilitation provider]

Finally, on the provider side, negative attitudes–particularly around disability–could hinder the provision of needed services. In resource-constrained settings, a few key informants noted, care may be deprioritized for individuals with HIV-related disability due to low expectations as some health care professions may “…think [a person with a disability] is going to die anyway. Tendency is to say ah can’t do anything” [Disabled People’s Organisation].

## Discussion

This study aimed to explore amongst relevant national- and local-level stakeholders in Zimbabwe awareness of HIV-related disability and perceived barriers to the provision and uptake of services for preventing, treating or managing HIV-related disabilities. While international actors, with input from some regional partners in sub-Saharan Africa are increasingly addressing HIV-related disability through advocacy and programmatic actions, less is known on how widely this information has been filtered down across the range of stakeholders working in the fields of HIV and disability at a national or local level. As these stakeholders are key for implementing responses to HIV-related disability, understanding the availability of specialised interventions and the perceptions of these stakeholders on barrier to uptake and provision of services is an important an under-researched area that this study seeks to fill. Our key finding is that while awareness of HIV-related disability is increasing among many key informants, there is a lag in concerted efforts to increase provision of coordinated, decentralized and accessible services that meet the needs of people living with HIV-related disabilities.

Establishing a coordinated, integrated response between rehabilitation and HIV service providers was cited as the best avenue for meeting the needs of people living with HIV-related disabilities. Early detection of HIV, prompt initiation and consistent adherence to ART and prevention/effective treatment of opportunistic infections reduces risk of developing or exacerbating disability [6, 9]. Meanwhile, rehabilitation can improve level of functioning, prevent deterioration of condition and find adaptations to mitigate activity and participation restrictions [14, 29]. Although most respondents believed Zimbabwe has strong systems for addressing disability and HIV separately, several areas for strengthening the response to HIV-related disability emerged.

First, more intensive, systematic screening for disability in people living with HIV is needed. The current process is more attuned to identifying obvious disabilities, as screening is done at the discretion of individual providers, who typically only suggest referral for the more noticeable disabilities. Subtle disabilities, particularly milder forms of cognitive impairment or developmental delay–which were identified in a recent systematic review as very strongly associated with HIV [9]—are thus less likely to be picked up and referred for follow-up. Other research by Hanass-Hancock & Alli (2015) from South Africa similarly found the lack of training of healthcare workers on the intersectionality between disability and HIV as well as the absence of disability screening tools to be an impediment to promoting access to needed rehabilitation services [30]. In addition to training of healthcare workers, increasing awareness among people living with HIV-related disabilities about their condition, strategies for management and available services was noted by key informants in this study and others [30, 31] as enabling comprehensive care.

Second, respondents noted that lack of integration creates barriers to provision of holistic care for people living with HIV-related disability. Most notably, HIV services often are not designed to accommodate the needs of people with disabilities, whether HIV-related or otherwise, a finding which has been gaining increasing attention in other research [31–34]. This situation may be changing as key informants noted, for example, the increasing engagement of DPOs to promote mainstreaming of disability within HIV prevention and care services, though funding gaps for needed resources remain. Furthermore, the fluctuating waves of illness and health associated with HIV can create disruptions in rehabilitation plans and providers may be ill-equipped at managing the unique multi-systemic, episodic nature of HIV-related disability. The episodic nature of disability also presents social, emotional and physical challenges for people living with HIV-related disability [35, 36]. For example, the inherent uncertainty of when periods of disablement will occur and how they will affect performance of daily activities and social participation, can lead to stress and difficulties planning for people with HIV-related disability [35].

Finally, the response to HIV-related disability is particularly lagging in rural areas. At the time of the study, there were no specific interventions for HIV-related disability, so individuals mostly had to navigate the rehabilitation and HIV sectors separately in an attempt to receive needed services. On the rehabilitation side, services are scarce in rural areas, so that people with disabilities have to travel prohibitively far distances to receive care. Costs associated with this travel were cited by almost all informants as one of the major barriers to accessing care. Chetty & Hanass-Hancock (2016) similarly noted centralisation, poorly-equipped centres and financial barriers as impeding access to rehabilitation for HIV-related disabilities in South Africa [31]. While community-based rehabilitation (CBR) has been successful in resource-poor settings as an alternative to hospital-based care, other studies have noted that for HIV-related disabilities there is still a need to increase integration with HIV services, which has thus far not been a core component of standard CBR [31]. On the HIV side, services for adults are generally more widespread but a major gap exists for infants in that there is only one lab in the country for early infant (<18 months) testing. Even for children above 18 months, ART coverage gaps remain high (41% vs. 77% for adults) due to factors such as the limited number of health centres equipped to deliver paediatric ART and difficulties administering the more complex paediatric drug regimens [37, 38]. The combination of delayed initiation of or inconsistent adherence to ART–which can increase likelihood of developing impairments–with prohibitively far and undersupplied rehabilitation could lead to worsening experiences of HIV-related disability, particularly for children in rural areas.

In interpreting the results of this study, some limitations are important to take into consideration. Notably, the small sample size (n = 10) may not reflect the experience and views of all relevant stakeholders in Zimbabwe. Although most interviewees represented organizations engaged in activities throughout Zimbabwe, as they were based in Harare, it is possible that their perspectives captured more the situation in the capital rather than that of the country at large. Furthermore, results of this case study may not be generalizable to the rest of sub-Saharan Africa, as contextual factors in Zimbabwe may vary from other places; notably Zimbabwe has a fairly well-developed rehabilitation system and high ART coverage, which other countries in the region may not benefit from. Additional studies from other contexts and with a wider range of stakeholders–most notably ones that focus on the voices of individuals living with HIV-related disabilities–is an urgent area for further research.

## Conclusion

As people living with HIV are surviving longer, HIV-related disabilities will become a major source of disability globally, particularly in sub-Saharan Africa where infection is widespread. Preventing, treating and managing HIV-related disabilities must become a key component of both HIV response efforts and rehabilitation strategies. While this research highlights that awareness among key stakeholders in HIV and disability is rising, steps to operationalize this knowledge and establish comprehensive, integrated interventions at the local level are lagging. In particular, there is a need to better understand how to incorporate disability prevention and rehabilitation interventions alongside HIV care and treatment strategies to adapt to the changing experiences of people living with HIV.

## Supporting information

S1 File “Semi-structured topic guide”. (DOCX)
