# Supplementary material for: Knowledge of HIV-related disabilities and challenges in accessing care: Qualitative research from Zimbabwe
Source: PLoS One. 2017 Aug 9;12(8):e0181144. doi: 10.1371/journal.pone.0181144 (PMC5549973; doi:10.1371/journal.pone.0181144)
Supplement: S1 File — (DOCX) [file pone.0181144.s001.docx]

| **Research interest**  Semi-Structured Interview Guide | **Interview questions** |
| --- | --- |
| 1. **Background on interviewee** | 1. How long have you been involved with [name of organization]? 2. What is your role in [name of organization]? |
| 1. **Background on organization (to be researched before interview, use to fill in gaps)** | *Will vary depending on organization* |
| 1. **Experience of HIV/AIDS in disability-focused organizations** | 1. Does the organization collect any information on the HIV status of clients? If so, are you able to give any indication of how many of your clients are living with HIV? 2. What, if any, disabilities do you observe amongst clients living with HIV? *(Probe: What disabilities are most commonly observed?)* In general, did these disabilities present before or after HIV infection? 3. From your experience, in what ways, if any, does HIV affect disability? (*Probe:* *Have you seen evidence of HIV/AIDS worsening pre-existing disabilities? Or the presentation of new disabilities that appear linked to HIV infection, disease progression, starting new ARVs, etc. Issues relating to double-stigma)* 4. What would you say are some of the greatest needs of people living with both HIV and disability? To what extent are these needs met in your client population? 5. What are some areas of unmet need for people who are living with both HIV and disability? What are some ways these could be addressed? What are some of the challenges/opportunities in addressing these needs? |
| 1. **Integration of HIV/AIDS in disability-focused organizations** | 1. What if any HIV services are on offer? How are they provided? *(Probes: referrals/provision of services directly etc.)* 2. If direct provision of service: What triggered the creation of these services? To what extent are they utilised by the targeted population? Do you feel they are adequate to meet the needs of this group? 3. If referrals: What is the extent of the linkages between your organization and these service providers? What prompted the collaboration? To what extent are these referral services used by the targeted population? Do you feel they are adequate to meet the needs of this group? 4. If organization is involved in rehabilitation: does the HIV status of individuals raise unique issues for the provision of rehabilitation services? (*Prompts:* in what ways? Episodic/changing vs. static disability) |
| 1. **Experience of disability in HIV/AIDS focused organizations** | 1. Does the organization collect any information on the prevalence of disability in your clients? If so, are you able to give any indication of how many of your clients are living with a disability? 2. What would you say are the most common disabilities affecting your clients? In general, did these disabilities present before or after HIV infection? 3. From your experience, in what ways, if any, does HIV affect disability? (*Probe:* *Have you seen evidence of HIV/AIDS worsening pre-existing disabilities? Or the presentation of new disabilities that appear linked to HIV infection, disease progression, starting new ARVs, etc. Issues relating to double-stigma)* 4. What would you say are some of the greatest needs of people living with both HIV and disability? To what extent are these needs met in your client population? 5. What are some areas of unmet need for people who are living with both HIV and disability? What are some ways these could be addressed? What are some of the challenges/opportunities in addressing these needs? |
| 1. **Integration of disability in HIV/AIDS focused organizations** | 1. Does your organization offer any services for the rehabilitation or treatment of disability? 2. If yes to (a): What specific services are offered? What triggered the creation of these services? 3. If yes to (a): To what extent are these services utilised by the targeted population? Do you feel they are adequate to meet the needs of this group? 4. Do you have any links with local NGOs, rehabilitation services or clinics for referring clients for the treatment/management of their disability? 5. If yes to (d): What is the extent of the linkages between your organization and these service providers? What prompted the collaboration? 6. If yes to (d): To what extent are these referral services used by the targeted population? Do you feel they are adequate to meet the needs of this group? |
| 1. **Implications for policy** | 1. Do you know of any efforts to address HIV/AIDS-associated disability through policy or specific programs in Zimbabwe? 2. If yes to (c), probes to elaborate: What is the current status of these efforts? What do they aim to accomplish? What have been some of the challenges in achieving them? 3. Do you think the issue of HIV/AIDS-associated disability needs to be addressed in local or national legislation/programs (e.g. National HIV/AIDS Strategic Plans)? 4. If yes, what specific issues to you feel should be prioritized? 5. From your experience working in this field, is HIV/AIDS-associated disability a recognized issue? If no, why do you think that is? |
| 1. **Other** | 1. Are there any other issues which I haven’t covered and which you feel are important.? |
